# Optimizing outcome reporting after robotic flexible ureteroscopy for management of renal calculi: Introducing the concept of tetrafecta

**DOI:** 10.1007/s11701-024-01858-1

**Published:** 2024-03-16

**Authors:** Morshed Salah, Mahmoud Laymon, Tawiz Gul, Hossameldin Alnawasra, Mohammed Ibrahim, Bela Tallai, Mohamed Ebrahim, Maged Alrayashi, Mohamed Abdelkareem, Abdulla Al-Ansari

**Affiliations:** 1 Urology Section, Surgery Department, Hazm Mebaireek General Hospital, Hamad Medical Corporation, Doha, Qatar; 2 Department of Surgery-Urology, College of Medicine, QU Health, Qatar University, Doha, Qatar; 3 Urology and Nephrology Center, Mansoura University, Mansoura, Egypt

**Keywords:** Robotic flexible ureteroscopy, RIRS, FURS, RFURS, Roboflex, Tetrafacta

## Abstract

Robotic flexible ureteroscopy (RFURS) has shown encouraging results in terms of stone free rate (SFR) and better ergonomics compared to conventional FURS. However, few studies have reported its outcomes. The goal of this study was to report our initial results of RFURS, furthermore we proposed a novel metrics for composite outcome reporting named tetrafecta. A retrospective analysis of electronic records of 100 patients treated with RFURS for renal stones between 2019 till 2023 was performed. Tetrafecta criteria included, complete stone removal after a single treatment session, without auxiliary procedures, absence of high-grade complications (GIII-V) and same-day hospital discharge. Mean patient age and stone size were 40.7 ± 9.2 and 11.7 ± 5.8 mm, respectively. Median stone volume was 916 (421–12,235) mm^3^. Twenty-eight patients had multiple renal stones. Staghorn stones were seen in 12 patients. Preoperative DJ stent was fixed in 58 patients. Median operative time and stone treatment time were 116 min (97–148) and 37 (22–69) min. The median stone treatment efficiency (STE) was 21.6 (8.9–41.6). A strong positive correlation between stone volume and STE (*R* = 0.8, *p* < 0.0001). Overall, 73 patients were stone free after the initial treatment session while tetrafecta was achieved in 70 patients. Univariate analysis showed that the stone size (*p* = *0*.008), acute infundibulopelvic angle (*p* = 0.023) and preoperative stenting (*p* = 0.017) had significant influence on achieving tetrafecta. Multivariate analysis identified preoperative stenting (OR 0.3, 95% CI 0.1–0.8, *p* = 0.019) as the only independent predictor of tetrafecta achievement. A comprehensive reporting methodology for reporting outcomes of RFURS is indicated for patient counseling and comparing different techniques. Tetrafecta was achieved in 70% of cases. Presence of significant residual stones ≥ 3mm was the leading cause of missing tetrafecta. Absence of preoperative stent was the only predictor of missing tetrafecta.

## Introduction

Urolithiasis is one of the most common urological disorders with increasing incidence and prevalence worldwide. Introduction of smaller caliber flexible ureteroscopes (FURS) and the use of high-power laser for lithotripsy have revolutionized the indications of retrograde intrarenal surgery (RIRS) for management of nephrolithiasis [1, 2]. However, several limitations have been reported with the use of FURS mainly related to ergonomics and prolonged radiation exposure [3–5]. Use of robotics in the field of endourology provided a significant comfort for the surgeon in terms of physical ergonomics [6]. RFURS using Avicenna Roboflex™ have shown encouraging results in terms of stone free rate (SFR) and better ergonomics compared to conventional flexible ureteroscopy [7, 8]. Nevertheless, there is a lack of standard definitions of the determinants of successful outcomes following FURS like the size of significant residual fragments, utilization of auxiliary procedures as ESWL to achieve complete stone clearance and number of treatment sessions. These disparities lead to heterogenous reporting of surgical outcomes and render comparison between different studies difficult. In this context we proposed a comprehensive set of criteria named “tetrafecta” that describes the successful achievement of four specific criteria aiming at optimizing outcome reporting after RFURS. Tetrafecta includes two surrogate measures of surgical adequacy: complete stone clearance in a single session without use of auxiliary procedures and two surrogate measures of patient safety: absence of high-grade complications and discharge on the same day of surgery. Therefore, the aim of this study is to investigate the incidence and predictors of achieving tetrafecta after RFURS for management of renal calculi.

## Patients and methods

### Patients

A retrospective analysis of a prospectively maintained database of 100 consecutive patients treated with RFURS for renal stones between October 2019 to May 2023 was performed. Preoperative patient assessment included detailed medical history, physical examination, urinalysis, urine culture, complete blood count, serum biochemistry and coagulation profile. Abdominal ultrasonography and computed tomography (CT) were performed in all patients. Stone characteristics including: number, localization, and volume were analyzed using the bone mode of the CT scan. Stone size was determined by measuring the longest axis and in cases of multiple calculi, stone size was defined as the sum of the longest axis of each stone. Stone volume was calculated using an ellipsoid formula: (Stone volume = 0.167 × *π* × *L* × *W* × *D*) where length (*L*), width (*W*), and depth (*D*) are stone diameter measured in three axes [9]. Renal infundibulopelvic angle (IPA) for lower calyceal stone was measured as an inner angle formed at the intersection of the ureteropelvic axis and the central axis of the lower pole infundibulum as defined by El-bahnasy et al. [10].

### Surgical technique

All procedures were performed under general anesthesia with the patient placed in lithotomy position. Initially, cystoscopy and a retrograde pyelography were done, then a ureteral access sheath (UAS) is placed over a guide wire under fluoroscopic guidance. Manual insertion of the ureteroscope into the UAS and then the ureteroscope is docked to the robotic arm covered by sterile plastic drape. Once the ureteroscope is placed correctly inside the renal pelvis, it can be steered from the console. The procedure started with inspection of the renal pelvicalyceal system for localization of the stones. Lithotripsy was performed using Holmium or Thulium fiber Laser (TFL). A combination of dusting, fragmentation, popcorning and basketing of the stone was attempted in all cases according to stone size, location and density. Stone clearance was assessed intraoperatively by direct inspection of the renal collecting system. A DJ was placed in all cases to be removed by outpatient cystoscopy.

### Operative parameters

The following intraoperative parameters were recorded: access time defined as the time interval between cystoscopy, retrograde pyelography, placement of access sheath and introduction of flexible ureteroscopy. Docking time defined as time needed to fix the sterile robotic arm to the scope. Console time started when the surgeon takes over the surgery including mapping of pelvicalyceal system, stone localization, laser lithotripsy and basketing of the stone. Stone treatment time was calculated as the sum of lasing and basketing times. Operative efficiency was calculated by dividing stone volume in mm^3^ by total operative time (minutes) while stone treatment efficiency (STE) was calculated by dividing stone volume in mm^3^ by stone treatment time [8].

### Postoperative care and follow up

All patients were kept in the postoperative care unit for monitoring and patients with uneventful postoperative course were discharged upon full recovery. All complications were stratified according to Dindo–Clavien system. Patients were instructed to follow up 1 month after the procedure. At the follow up visit, non-contrast CT scan was done to assess SFR that was classified into three grades: Grade A (no residual fragments (RF) on CT scan), Grade B (RF ≤ 2mm) and Grade C (RF > 2mm).

### Outcome measures

The primary outcome was to report the rate and predictors of achieving tetrafecta following RFURS. Tetrafecta was defined as complete stone clearance (no RF) after a single treatment session, no auxiliary procedures, absence of high-grade complications (GIII-V) and same-day hospital discharge. Patients who met all these combined criteria were considered to have achieved RFURS tetrafecta. Patients’ stone and perioperative characteristics were compared based on achievement of tetrafecta. The secondary outcome was to assess the predictive capacity of a well-established R.I.R.S scoring system to predict tetrafecta [11].

### Statistical analysis

Continuous variables were expressed as the mean ± SD or median (range) and categorical data were presented by *n* (%). Continuous variables were analyzed using Student’s *t* or Mann–Whitney *U* tests. Categorical data were analyzed by Chi-square. Next, multivariate analysis using a logistic regression model in a stepwise method was performed to identify independent factors of tetrafecta achievement. The strength of the relationship between variables was determined using Spearman’s correlation, with significance set at 0.05. The predictive ability of the R.I.R.S. scoring system was evaluated by the area under the receiver operating characteristic (AUROC) curve. Statistical significance was set at *p* < 0.05. The analysis was performed with the Statistical Package for Social Sciences, version 11.5 (SPSS, IBM, Armonk, NY). Statistical significance was considered at *p* < 0.05.

## Results

### Patient and stone characteristics

A total of 100 consecutive patients were included in this study. Mean patient age was 40.7 ± 9.2, associated comorbidities were seen in 27 patients and a single patient suffered ischemic heart disease and was maintained on anticoagulants that was not stopped before surgery. Mean stone size was 11.7 ± 5.8 mm. Median stone volume was 916 (421–12,235) mm^3^. Twenty-eight patients had multiple renal stones while 26 patients harbored a single calyceal stone. Partial staghorn stones were seen in 12 patients, lower pole stones were found in 41 patients while 47 patients had a stone in the renal pelvis. The mean renal IPA was 52.7 ± 16.6 and it was less than 30 in 5 patients. Three patients with anomalous kidneys were identified. Patient demographics and stone characteristics are summarized in Table 1**.**

**Table 1:** Demographics and preoperative characteristics of 100 patients treated with RFURS between October 2019 till May 2023

Parameter	Total	Tetrafecta		*p* value
		Achieved	Not achieved	
Number of patients	100	70	30	
Age (mean ± SD)	40.7 ± 9.2	39.9 ± 8.5	42.6 ± 10.8	0.19
Laterality				0.1
Right	54	41 (58.6%	13 (43.3%)	
Left	46	29 (41.4%)	17 (56.7%)	
Hypertension (yes)	15	12 (17%)	3 (10%)	0.3
Diabetes mellitus (yes)	12	11 (15.7%)	1 (3.3%)	0.08
BMI (Median, IQR)	28.4 (26.4–32)	28.4 (26.8–31.2)	29.6 (26–32)	0.9
Serum creatinine (mean ± SD)	0.9 ± 0.3	0.8 ± 0.2	1.1 ± 0.3	0.3
Preoperative urine culture				0.5
Negative	94	65 (92.9%)	29 (96.7%)	
Positive	6	5 (7%)	1 (3.3%)	
Preoperative DJ stent				0.017
No	42	24 (34.3%)	18 (60%)	
Yes	58	46 (65.7%)	12 (40%)	
Preoperative hydronephrosis (yes)	29	19 (27%)	10 (33.3%)	0.5
Associated renal congenital anomalies	3	1 (1.4%)	2 (6.7%)	0.1
Horse-shoe kidney	1	1	0	
Ectopic pelvic kidney	1	0	1 (3.3%)	
Polycystic kidney	1	0	1 (3.3%)	
Number of stones				0.8
Single	72	50 (71.4%)	22 (73.3%)	
Multiple	28	20 (28.6%)	8 (26.7%)	
Stone location				0.6
Renal pelvis	47	33 (47.1%)	14 (46.7%)	
Calyceal	41	30 (42.9%)	11 (36.7%)	
Renal pelvis and calyceal	12	7 (10%)	5 (16.7%)	
Lower pole stones (yes)	41	28 (40%)	13 (43.3%)	0.7
RIPA^1^ (mean ± SD)	52.7 ± 16.6	55.5 ± 14.9	44.4 ± 19.3	0.08
RIPA < 30	5	1(1.4%)	4 (13.3%)	0.04
RIL^2^ in mm (mean ± SD)	38.4 ± 11.8	39.3 ± 11.9	35.6 ± 11.6	0.4
RIW^3^ in mm (mean ± SD)	13.4 ± 6.4	13.8 ± 7	11.3 ± 4.8	0.3
Stone volume in mm^3^ (median, IQR)	916 (421–1451)	898 (420–1347)	972 (406–2912)	0.2
Stone width in mm (mean ± SD)	11.7 ± 5.8	10.8 ± 3.7	13.8 ± 8.6	0.008
Stone surface area in mm^2^(median, IQR)	170 (110–244)	170 (112–225)	169 (81–304)	0.8
Hounsfield unit (mean ± SD)	1059 ± 293	1056 ± 289	1114 ± 331	0.3

^1^Renal infundibulopelvic angle, ^2^renal infundibular length, ^3^renal infundibular width

### Operative findings

Preoperative DJ stent was fixed in 58 patients. Mean access and docking times were 12.3 ± 7.4 and 7.8 ± 3.2 min, respectively. Median stone treatment time was 37 (22–69) min. The median operative time was 116 min (IQR 97–148). The median STE was 21.6 (8.9–41.6). A strong positive correlation between stone volume and STE (Spearman’s rho = 0.8, *p* < 0.0001). Among patients with stone volume larger than 2500 mm^3^, the median STE was 40.8 (38–151) mm^3^/min. Holmium and TFL were utilized in laser was used in 60 and 40 patients, respectively with no significant differences between both in terms of lasing time, laser energy and STE. The median length of hospital stay was 9.3 h (5.4–166). Only, 5 patients were admitted for one day and the remaining 95 patients were discharged home at the same day of surgery. Operative findings are detailed in Table 2.

**Table 2:** Perioperative parameters of 100 patients treated with RFURS between October 2019 till May 2023

Parameter	Total	Tetrafecta		*p* value
		Achieved	Not achieved	
Number of patients	100	70	30	
LASER type				0.6
Holmium	60	41 (58.6%)	19 (63.3%)	
Thulium fiber laser (TFL)	40	29 (41.4%)	11 (36.7%)	
Total laser energy (KJ) (median)	17.5 (6.3–45)	16.2 (6.2–34)	30.8 (5.5–55)	0.6
Operative time in min (median, IQR)	116 (97–148)	115 (97–145)	123 (97.5–152)	0.4
Access time in min (±SD)	12.3 ± 7.4	12.3 ± 2	12.2 ± 2.1	0.8
Docking time in min (±SD)	7.8 ± 3.25	6.8 ± 1.2	6.6 ± 1.3	0.4
Laser time in min (median, IQR)	30 (15–60)	30 (15–60)	20 (15–61)	0.5
Basketing time in min (±SD)	8.7 ± 1.8	8.7 ± 1.8	8.8 ± 2.1	0.8
Stone treatment time in min (median, IQR)	37 (22–69)	37 (22–67)	31 (24–71)	0.15
Stone treatment efficiency in mm^3^/min (median)	21.6 (8.9–41.6)	21.6 (8.8–41.6)	28.7 (8.9–47)	0.78
Operative efficiency in mm^3^/min (median)	7 (4–10.7)	6.9 (4.1–10.3)	7.2 (4.3–26)	0.45
Retreatment rate	13	0	13 (43.3%)	
Retreatment procedures			Flexible URS (7) ESWL (5) Semi-rigid URS (1)	

Overall, 73 patients were stone free (Grade A, no RF) after the initial treatment without auxiliary procedures. Of the 27 patients with RF, 5 had RF ≤ 2mm (Grade B) and 22 had RF > 2mm (Grade C). Eight patients underwent a second RIRS procedure, 5 were treated with ESWL and the remaining 14 were managed conservatively. A total of eight complications were recorded in five patients, including ureteral injury with extravasation of contrast in one, urinary tract infection in two, fever in two, stent related discomfort in two and haematuria in two patients. No significant difference between SF and non-SF groups regarding occurrence of postoperative complications (*p* = 0.2). All patients were treated conservatively with analgesics, anticholinergics and/or prolonged double-J stent placement in the ureter for as long as 4 weeks. Postoperative complications are shown in Table 3**.**

**Table 3:** Perioperative complications after RFURS stratified by Dindo–Clavien system

Complication	Number	Management	Dindo–Clavien classification
Haematuria	1	Hemostatics, bladder irrigation	I
Fever	2	Antipyretics	I
Stent-related discomfort	2	Analgesics, anticholinergics	I
Urinary tract infection (UTI)	2	Antibiotics	II
Ureteric injury with extravasation	1	DJ stent fixation	III

### Predictors of tetrafecta after RFURS

Tetrafecta was achieved in 70 patients. Complete stone clearance in a single session, absence of high-grade complication and same-day hospital discharge were obtained in 73, 99 and 95 patients, respectively. Presence of significant residual stones ≥ 3mm was the leading cause of missing tetrafecta. Univariate analysis showed that the stone size (*p* = *0*.008), lower pole IPA < 30 (*p* = 0.023) and preoperative stenting (*p* = 0.017) had significant influence on achieving tetrafecta after RFURS. Multivariate logistic regression analysis using all factors showed that only preoperative stenting **(**OR 0.3, 95% CI 0.1–0.8, *p* = 0.019) was independent predictor of tetrafecta achievement following RFURS.

### Validation of R.I.R.S nomogram to predict Tetrafecta

**Fig. 1:**
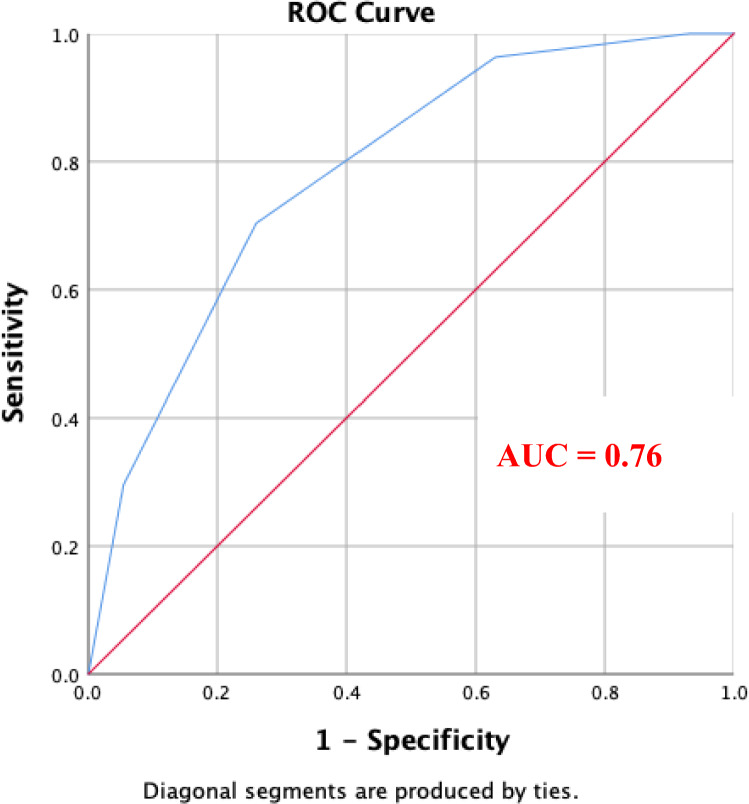
ROC curve with area under the curve (AUC) for the role of RIRS scoring system in predicting tetrafecta after robotic flexible ureteroscopy (FURS)

The R.I.R.S. score was significantly higher for those who did not achieve tetrafecta (7 ± 1. 08 vs 5.9 ± 1.04, *p* < 0.0001), respectively. Area under the curve (AUC) was 0.76, CI 0.65–0.86, *p* < 0.0001), Fig. 1**.** ROC curve analysis showed that RIRS could predict SFR with cut off value of 6.5 with sensitivity 72% and specificity 77%.

## Discussion

Adoption of a standardized reporting methodology of the outcomes after surgical procedures is mandatory for better characterization of surgical morbidity, comparing different surgical techniques and institutional experience. In this context, a composite outcome reporting methods named trifecta and pentafecta have been employed in various fields of urology including urologic oncology and many endourologic procedures [12–14]. In this study, we proposed a new metrics for reporting outcomes after RFURS named tetrafecta, including complete stone removal after single session, absence of high-grade complication and hospital discharge in the same day of surgery. The first two criteria are surrogate of surgical adequacy and the last two are surrogate of patient safety.

Several important findings were seen in this study. First, the rate of tetrafecta achievement was 70% and presence of residual stones was the leading cause of missing tetrafecta. Residual stone fragments ≥ 3 mm were considered as significant residuals and we found that SFR at 1 month was 73% and retreatment rate was 13%. In a randomized controlled trial comparing conventional vs RFURS, Gevelate et al. reported a SFR of 92.4% among 66 patients treated with RFURS, but the follow up radiologic tool whether CT scan or plain X ray was not clearly mentioned [7]. Klein et al. documented SFR reaching 90% among 250 patients managed with RFURS as detected by ultrasonography and KUB before stent removal [8]. The higher incidence of residual stone fragments in our series could be attributed to utilization of NCCT in all patients during follow up. It has been proven that NCCT has higher sensitivity than KUB and US in detection small residual fragments [15].

On multivariate analysis, we identified stone size and lower pole IPA < 30 as the only significant predictors of RF. Stone free rate was 80% and 44% for renal calculi ≤ 10 mm and > 20 mm, respectively (*p* = 0.043). Breda et al. analyzed the outcomes of FURS for management of multiple intrarenal calculi and found that SFR was achieved in 65% and 92% after first and second procedure, respectively [16]. Hussain et al. reported a SFR of 96.5% and 58.3% in patients with stones < 2 cm and > 2 cm, respectively [17]. In the present study, lower calyceal stones were found in 41 patients and no difference was encountered regarding SFR between lower, middle, and upper calyceal groups and these results are in accordance with previous studies that confirmed the safety and efficacy of FURS for lower pole stones [18, 19]. Nevertheless, Lower pole IPA < 30 was associated with higher residual stones that can be explained by limited deflection of FURS rendering access to the lower calyx difficult [20, 21].

The stone burden in this study varied significantly with evolution of the learning curve and included a wide range of stone shapes, sizes and composition, starting from small stone at the beginning of learning curve till partial staghorn stone. The median stone volume was 916 (421–12,235) mm^3^ which is comparable to many previous series reporting the results of flexible ureteroscopy [22–24]. STE was strongly correlated with stone volume with a median of 15.4 mm^3^/min for stone volume < 2500 mm^3^ and reaching 40.8 mm^3^/min for stone volume > 2500 mm^3^. Our findings were consistent with previous publications, Klein et al. reported a STE of 33 mm^3^/min with median stone volume of 1798 mm [8]. Hyams et al. reported a STE of 98 mm^3^/min while using flexible ureteroscopy for large renal stones ranging between 2 and 3 cm [25].

It is widely accepted that routine preoperative stenting is not recommended because of increased risk of radiation exposure, anesthesia and cost [26]. The current study proved that prestenting was associated with higher incidence of SFR and tetrafecta achievement on multivariate analysis, a finding that is consistent with a lot of published data. In a large retrospective study including more than 6500 patients, Chai et al. [27] investigated the impact of prestenting on outcomes of RIRS and found longer operative time and higher incidence of fever and sepsis among the non pre-stented patients. Several studies have shown that preoperative stenting for 1–2 weeks may allow passive dilatation of the ureter, increasing the success of ureteric access sheath (UAS) placement and reducing the risk of high‐grade ureteric injuries [28]. However, all previously mentioned studies are limited by their retrospective design. Based on these published data, we recommend a tailored approach and proper counseling for each patient, as ureteral stenting can be mandatory preoperatively for pain relief and drainage of obstructed infected kidneys or intraoperatively for dilatation of tight ureter when passage of ureteral access sheath is not possible. A well-designed and adequately-powered RCT to address the impact of preoperative stenting is warranted.

Postoperative complications were seen in five patients and the vast majority of complications were low grade (GI, II). Ureteric injury with contrast extravasation was seen in a single case. Previous studies showed that the incidence of complications after RFURS did not differ from conventional one and majority were low grade [29]. Our study is unique in that 95% of patients were discharged safely on the same day of surgery with median hospital stay of 9.3 h. Patients were kept in the post-anesthesia care unit for 0.5–1 h for monitoring followed by an additional 4–6 h of recovery in the same-day surgery unit. In previously published studies, the median hospital stay ranged between 1 and 1.5 days [8, 25, 30]. Day-case surgery is associated with early ambulation of the patient reducing the risk of thromboembolic complications, decrease nosocomial infections, reducing hospital costs and improve bed turn over.

Finally, our study is not devoid of limitations and drawbacks. First, the study is retrospective single center with relatively small number of patients and inherent to all drawbacks with such study design. Second, the number of large and branching stones were small because this study represents the initial experience and evolution of the surgeons’ learning curve. Nevertheless, data-reporting outcomes of RFURS are sparse in urologic literature and this study provides a new concept for reporting outcomes of RFURS named tetrafecta aiming at achieving optimal reporting methodology and provides an arm for comparing outcomes between different institutions and different techniques. Further prospective studies evaluating the role of RFURS in management of large renal stones are highly recommended.

## Conclusion

A comprehensive reporting methodology for reporting outcomes of RFURS is highly indicated for patient counseling and comparing different techniques. The incidence of tetrafecta after RFURS was 70% and presence of residual stones was the commonest cause of missing tetrafecta. On univariate analysis, larger stone size, acute lower pole IPA and absence of preoperative stenting were significant predictors of missing tetrafecta. Multivariate analysis identified preoperative stent as the only predictor of achieving tetrafecta.

## Data Availability

No datasets were generated or analyzed during the current study.
